# Recombinant Sj16 protein with novel activity alleviates hepatic granulomatous inflammation and fibrosis induced by *Schistosoma japonicum* associated with M2 macrophages in a mouse model

**DOI:** 10.1186/s13071-019-3697-z

**Published:** 2019-09-23

**Authors:** Jia Shen, Lifu Wang, Mei Peng, Zhen Liu, Beibei Zhang, Tao Zhou, Xi Sun, Zhongdao Wu

**Affiliations:** 1 Department of Parasitology of Zhongshan School of Medicine, Sun Yat-sen University, Guangzhou, 510080 Guangdong China; 2 Key Laboratory of Tropical Disease Control (SYSU), Ministry of Education, Guangzhou, 510080 Guangdong China; 3 Provincial Engineering Technology Research Center for Biological Vector Control, Guangzhou, 510080 Guangdong China

**Keywords:** *Schistosoma japonicum*, rSj16, Granulomatous inflammation, Fibrosis, M2 macrophages

## Abstract

**Background:**

Potent granulomatous inflammation responses induced by schistosome eggs and resultant fibrosis are the primary causes of morbidity in schistosomiasis. Recombinant Sj16 (rSj16), a 16-kDa protein of *Schistosoma japonicum* produced in *Escherichia coli*, has been demonstrated to have novel immunoregulatory effects *in vivo* and *in vitro*. Thus, this study investigated the anti-inflammatory and anti-fibrotic effects of rSj16 treatment in *S. japonicum-*infected mice and demonstrated the immune modulation between the schistosome and the host.

**Methods:**

*Schistosoma japonicum* infected mice were treated with the rSj16 protein and Sj16 peptide at different time points post-infection to assess their efficacy at the optimal time point. Sj16 peptide and/or Praziquantel (PZQ) treatments were initiated at week 5 post-infection to compare the therapeutic efficacy of each regimen. Hepatic granulomatous inflammation, fibrosis and cytokine production (pro-inflammatory, Th1, Th2, Th17 and regulatory cytokines IL-10) were detected. Moreover, M2 macrophages were measured to illuminate the mechanisms of Sj16.

**Results:**

The rSj16 protein and Sj16 peptide had significant protective effects in *S. japonicum*-infected mice, as shown by decreased granuloma formation, areas of collagen deposition and inhibition of pro-inflammatory Th1, Th2 and Th17 cytokine production. These protective activities were more obvious when animals were treated with either the Sj16 protein or peptide at early stages post-infection. Interestingly, the combined treatment of PZQ and Sj16 was more effective and upregulated IL-10 production than administration of PZQ alone in infected mice. Furthermore, the Sj16 treatment alleviated the pathological effects associated with activated M2 macrophages.

**Conclusions:**

This study demonstrates the anti-inflammatory and anti-fibrotic effects of rSj16 in schistosomiasis. Therefore, the combination of rSj16 with PZQ could be a viable and promising therapeutic strategy for schistosomiasis. In addition, this investigation provides additional information on schistosome-mediated immune modulation and host-parasite interactions.

## Background

Schistosomiasis, which is caused by blood flukes (trematodes) of the genus *Schistosoma*, is still a significant public health problem in tropical and subtropical areas in 78 different countries [1]. Schistosomiasis is described as the third most devastating tropical disease in the world, after malaria and intestinal helminthiasis [2, 3]. It is estimated that in 2017, at least 220.8 million people required preventive treatment for schistosomiasis, out of whom more than 102.8 million people were reported to receive treatment [1]. The pathogenic mechanism of schistosomiasis is primarily attributed to helminth egg-induced granulomas in different organs and tissues and secondary fibrosis [4]. In intestinal schistosomiasis, which is mostly caused by *Schistosoma mansoni* and *Schistosoma japonicum,* egg granuloma formation mainly occurs in the liver and the intestinal wall, leading to fibrosis and advanced disease, such as portal hypertension and hepatosplenomegaly. Two serious complications, including ascites and variceal bleeding of schistosomiasis, can result in the death of the patient [4–6]. Compared with schistosomiasis mansoni, the liver pathology of schistosomiasis japonica is more serious, with more egg granulomas (deposited eggs as clusters) with larger sizes and more extensive diffuse inflammatory infiltration [7, 8]. In urogenital schistosomiasis caused by *S. haematobium,* egg deposition and granuloma formation mainly occur in the urinary bladder wall, which results in hematuria, dysuria and further complications, such as bladder calcification and urinary tract fibrosis, which causes obstructive uropathy and bladder malignancies [9]. Additionally, a large percentage of women and men with urogenital schistosomiasis acquire genital lesions and other complications, such as irreversible infertility [10].

Various types of immune responses are involved in regulating immunopathogenesis of schistosomiasis. For example, the balance of T helper 1 cell (Th1) and Th2 cytokines determines hepatic granulomatous inflammation [11, 12]. Th17 responses have been linked with severe hepatic inflammation and fibrosis [13], and immunomodulatory cytokines (IL-10, TGF-β) have been shown to inhibit the immunopathology of schistosomiasis [11, 12]. Macrophages are one of the main cellular constituents of granulomas [14]. It has been well demonstrated that tissue macrophages have considerable plasticity and can quickly switch their function in response to different environmental stimuli by changing their phenotypes, which are described as classically activated macrophages (M1 macrophages) and alternatively activated macrophages (M2 macrophages) [15, 16]. M1 macrophages mediate the initial inflammatory response, whereas M2 macrophages are induced in response to the Th2 cytokines IL-4 and IL-13 [17] to exhibit potent immunoregulatory activity [18], such as suppressing pro-inflammatory Th1 and Th17 responses, which contribute to tissue injury [19, 20]. Macrophages are recognized to play an important role in regulating granulomatous inflammation and fibrosis during schistosomiasis [21–25].

Praziquantel (PZQ) is currently the best therapeutic choice for the treatment of schistosomiasis and has been widely used for several decades [26]. PZQ acts by inducing surface damage of adult worms that causes the parasite to detach from venous walls and die [27]. In general, parasites are cleared after administered efficacious anti-parasitic drugs and subsequent fibrosis is slightly reduced due to the elimination of worms [28]. However, continuous aggravation of hepatic granulomatous inflammatory responses and subsequent fibrosis are commonly observed in some patients [29]. It is well known that granuloma inflammation and subsequent fibrosis are the primary cause of death in schistosomiasis, so it is important to prevent their progress.

The recombinant protein Sj16 (rSj16), a 16 KDa protein of *S. japonicum* produced in *Escherichia coli*, has been demonstrated to have definitive anti-inflammatory effects *in vitro* and *in vivo* by our group [30–35]. *In vitro*, rSj16 has been shown to decrease lipopolysaccharide (LPS)-induced pro-inflammatory cytokine production and to increase the levels of the immunoregulatory cytokine IL-10 in dendritic cells and RAW264.7 macrophages [30, 31]. *In vivo*, rSj16 has been reported to reduce the severity of complete Freundʼs adjuvant-induced arthritis in a rat model [32] and to protect against dextran sulfate sodium-induced colitis in mice [33]. Furthermore, rSj16 can suppress the recruitment of thioglycollate-mediated leukocytes to the peritoneal cavity of mice [34]. Based on these observations, we were interested in testing the anti-inflammatory and anti-fibrotic effects of rSj16 alone and in combination with the anti-parasitic PZQ in *S. japonicum-*infected mice. Hepatic granulomatous inflammation, fibrosis and cytokine production (pro-inflammatory, Th1, Th2, Th17 and regulatory cytokines IL-10) were detected.

## Methods

### Animals and drugs

Six 8-week-old (20–25 g) male Bagg albino (BALB/c) mice were purchased from the Laboratory Animal Center of Sun Yat-Sen University, Guangzhou, China. Animals were housed under specific-pathogen-free conditions, were group housed in ventilated cages in a temperature controlled room (25 °C) under a 12:12 h light/dark photocycle and were fed standard mouse chow, and had access to water *ad libitum*. PZQ was obtained from the Center for Disease Control and Prevention of China as tablets and dispersed in water after pulverization, and then administered by oral gavage [36, 37].

### Preparation of recombinant proteins (rSj16 and glutathione S-transferase)

Recombinant Sj16 and 28 KDa glutathione S-transferase (GST) were produced as previously described [33, 38]. Briefly, the recombinant plasmid pGEX-4 T-1-Sj16 was constructed and transformed into *E. coli* BL21. rSj16 was expressed as a GST-Sj16 fusion protein and cleaved with thrombin (Sigma-Aldrich, St. Louis, Missouri, USA) after purification with a GSTrap^TM^ FFresin column (Amersham Pharmacia, Piscataway, NJ, USA). rSj16 and GST were separately collected from the column and purified by sodium dodecyl sulfate-polyacrylamide gel electrophoresis (SDS-PAGE). The endotoxins of the proteins were removed by treatment with Affinity PakDetoxi-Gel Endotoxin Removing Gel (Thermo Fisher Scientific, Waltham, MA, USA). The protein concentration was determined using the Pierce BCA Protein Assay Kit (Thermo Fisher Scientific, Waltham, MA, USA).

### Production of Sj16 peptide

In a previous study, we reported that Sj16 contains a functional N-terminal nuclear localization signal [39]. A section of the key amino acid sequence of this N-terminal nuclear localization signal, krsfrkgrhhiykvmdkyirk, was synthesized as Sj16 peptide. Production of Sj16 peptide was performed by Guangzhou Tera Biological Technology Co., Ltd. (Guangzhou, China), and Sj16 peptide was obtained a purity of > 98%. The synthesized powder of Sj16 peptide was dispersed in PBS solution.

### Parasite infections

Six 8-week-old male BALB/c mice were percutaneously infected with 20 cercariae of *S. japonicum* (Chinese mainland strain) obtained from infected *Oncomelania hupensis* snails purchased from the Jiangsu Institute of Parasitic Diseases (Wuxi, China).

### Experimental design

**Fig. 1:**
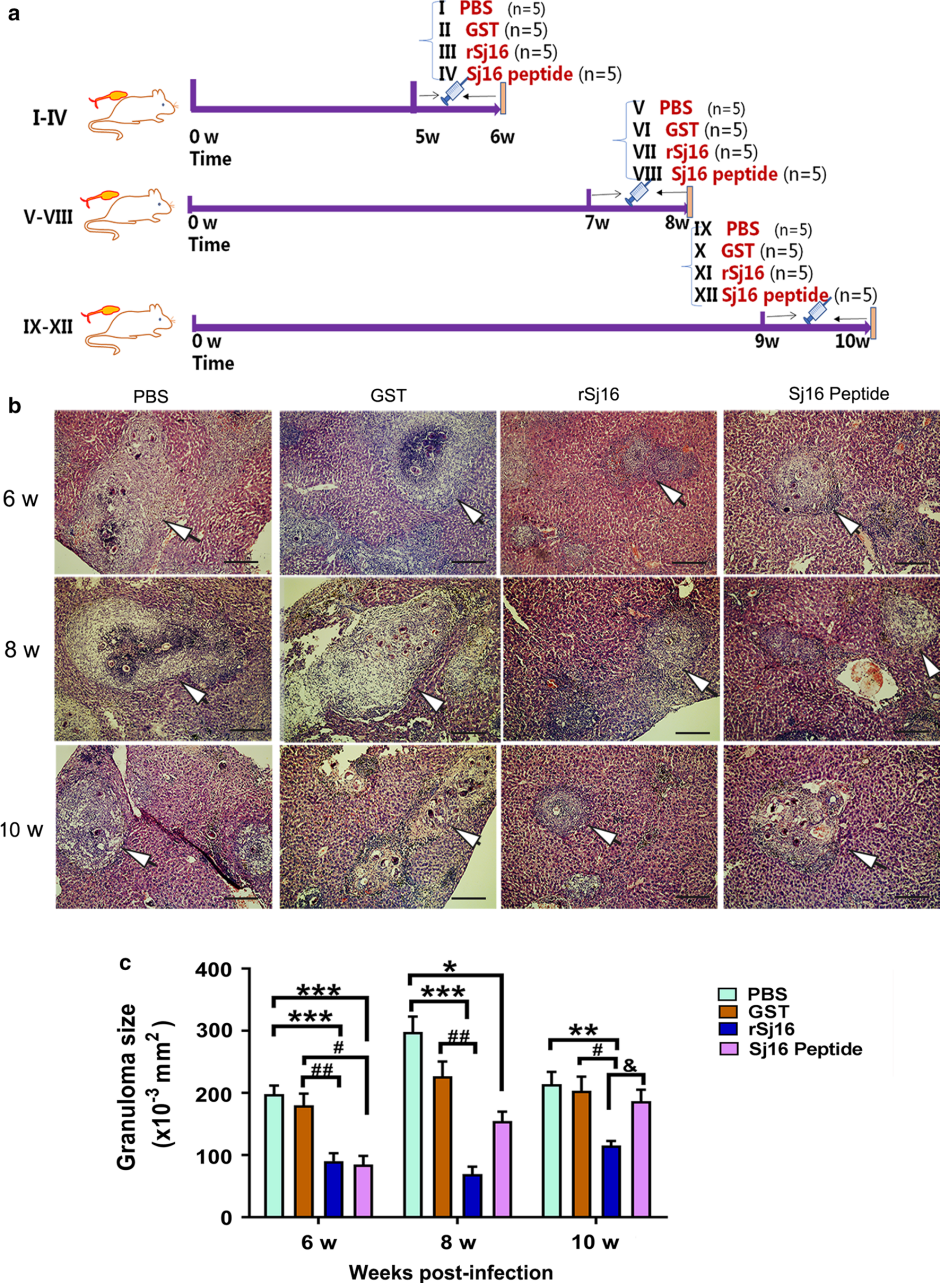
rSj16 protein and Sj16 peptide alleviated hepatic granuloma in *S. japonicum* infected mice. **a** The experiment flow chart. Infected BALB/c mice were respectively injected with PBS, GST protein (50 µg/d), rSj16 (50 µg/d), Sj16 peptide (50 µg/d) for 7 consecutive days before the animals sacrificed at 6, 8 and 10 weeks post-infection. **b** H&E staining of representative hepatic granulomas. *Scale-bars*: 100 μm. **c** Size of liver granulomas. Results are expressed as the mean ± SEM of 4–5 mice per group. *P < 0.05, **P < 0.01, ***P < 0.001, compared with the PBS group; ^##^*P* < 0.01, ^###^*P* < 0.001, compared with the GST group; ^&^*P* < 0.05, the rSj16 group compared with Sj16 peptide group. Similar results were obtained in two repeat experiments

Two sets of experiments were performed. In the first set of experiments, *S. japonicum*-infected mice were divided into 12 groups. Infected mice from groups (i), (ii), (iii) and (iv) were intraperitoneally (i.p.) injected with sterile PBS (100 µl/d), the GST protein (50 µg/d), the rSj16 protein (50 µg/d), and Sj16 peptide (50 µg/d), respectively, for 7 consecutive days starting from week 5 post-infection and were sacrificed at week 6 post-infection. Infected mice from groups (v), (vi), (vii) and (viii) were i.p. injected with sterile PBS (100 µl/d), the GST protein (50 µg/d), the rSj16 protein (50 µg/d), and Sj16 peptide (50 µg/d), respectively, for 7 consecutive days starting from week 7 post-infection and were sacrificed at week 8 post-infection. Infected mice from groups (ix), (x), (xi) and (xii) were i.p. injected with sterile PBS (100 µl/d), the GST protein (50 µg/d), the rSj16 protein (50 µg/d), and Sj16 peptide (50 µg/d), respectively, for 7 consecutive days starting from week 9 post-infection and were sacrificed at week 10 post-infection. Five mice were assigned to each of the above groups (Fig. 1a).

In the second set of experiments, *S. japonicum*-infected mice were divided into 4 groups. Infected mice from group (i) were i.p. injected with sterile PBS (100 µl/d for 5 weeks) starting from week 5. Infected mice from group (ii) were administered PZQ (150 mg/kg/day for 3 days) by an intragastric injection starting from week 5. Infected mice from group (iii) were i.p. injected with Sj16 peptide (50 µg/d for 5 weeks) starting from week 5. Infected mice from group (iv) were treated with both PZQ and Sj16 peptide at the same individual drug doses and on the timetable described for groups (ii) and (iii). Six mice were assigned to each group and sacrificed at 10 weeks post-infection (Fig. 4a).

### Histopathology, fibrosis and immunofluorescence

Liver tissues were fixed in 4% neutral buffered formalin, embedded in paraffin for sectioning, dewaxed and stained with hematoxylin and eosin (H&E) for granuloma analysis or with Masson’s trichrome for fibrosis analysis. For Massonʼs trichrome analysis, collagen is shown as blue in Massonʼs staining, and the blue area reflects the amount of collagen. We distinguished the severity of fibrosis by the area of collagen. The extent of granulomatous inflammation around eggs was measured by computer-assisted morphometric analysis using Image-Pro Plus software (Media Cybernetics, Rockville, MD, USA) as previously described [40]. At least 20 granulomas were measured per liver in 4–6 individual mice per group. Data are expressed in area units. For immunofluorescence analysis, the liver sections were boiled in 10 mmol/l citrate buffer (pH 6.0) for 15 min in a microwave oven for epitope retrieval. After blocking with 5% bovine serum albumin for 60 min, the sections were incubated with a primary antibody (anti-mouse Arg and F4/80 (Biotin-labelled)) (Abcam, Cambridge, UK) overnight at 4 °C. The sections were washed three times with PBS, incubated with a fluorescein isothiocyanate (FITC)-conjugated secondary antibody (Arg detection) for 60 min at room temperature, and stained with 4,6-diamidino-2-phenylindole (DAPI). Immunofluorescence images were captured by fluorescence microscopy (Leica, Frankfurt, Germany). For immunohistochemistry analysis, the de-waxed sections were washed three times in PBS and boiled in 10 mmol/l citrate buffer for 20 min in a microwave oven for epitope retrieval. After slow cooling and washing with PBS, the sections were incubated in 3% hydrogen peroxide for 10 min and washed. Then, 1% BSA was used to block the sections for 1 h at room temperature. Primary antibodies against IL-10 (GB11110, Wuhan Servicebio Technology CO., Ltd., Wuhan, China, used at a 1:400 dilution) and TGF-β (GB11179, Wuhan Servicebio Technology CO., Ltd., Wuhan, China, used at a 1:500 dilution) were incubated overnight at 4 °C. After washing three times with PBS, one drop of a ready-to-use HRP labelled anti-mouse and anti-rabbit general secondary antibody (DAKO, Copenhagen, Denmark) was used to incubate each section for 45 min at room temperature. Then, the sections were carefully monitored after 50 μl of substrate was added. Finally, to study the nuclear structure, the sections were counterstained with hematoxylin, dehydrated and covered with neutral gum, and examined under a microscope.

### Cytokine analysis

The cytokines IL-6, TNF-α, IL-17, IFN-γ, IL-4 and IL-10 in serum and the culture supernatant were assayed using a Cytometric Bead Array (CBA) kit (BD Biosciences, San Jose, CA, USA) according to the manufacturer’s instructions. Data were acquired on a FACScan flow cytometer (Beckman Coulter, Fullerton, CA, USA) and analysed using Kaluza software (Beckman Coulter). The cytokine concentrations were calculated by standard curves.

### Macrophage isolation and culture

Peritoneal macrophages were isolated as previously described [41]. Briefly, 6–8 week-old BALB/c mice were euthanized by CO_2_ asphyxiation and immersed in 75% alcohol for 2–5 min. Five millilitres of cold PBS containing penicillin (100 U/ml) and streptomycin (100 mg/ml) was injected into the abdominal cavity of the mouse and recollected after rubbing the abdomen for several seconds. The harvested suspended cells were transferred to sterile centrifuge tubes and centrifuged at 250×*g* for 10 min at 4 °C. The cells were resuspended in complete RPMI-1640 medium (GIBCO Laboratories, Grand Island, NY, USA) with 10% foetal bovine serum (FBS; GIBCO Laboratories) containing penicillin (100 U/ml) and streptomycin (100 mg/ml), counted and seeded into 12-well culture plates (1 × 10^6^ cells/well). After culturing at 37 °C with 5% CO_2_ for 2 h, the wells were washed with sterile PBS three times to remove non-adherent cells, and fresh complete RPMI-1640 medium was added. Macrophages were collected after stimulation with lipopolysaccharide (LPS) (1 μg/ml; Sigma-Aldrich), GST (10 μg/ml), rSj16 (10 μg/ml), Sj16 peptide (10 µg/ml), LPS (1 μg/ml) + GST (10 μg/ml), LPS (1 μg/ml) + rSj16 (10 μg/ml), LPS (1 μg/ml) + Sj16 peptide (10 µg/ml), or medium alone for 24 h.

### Flow cytometry

Liver leukocytes of infected mice were prepared by Ficoll-Hypaque (Hao Yang Biological Manufacture, Tianjin, China) gradient centrifugation after being dispersed through a 70 µm nylon strainer. For flow cytometry analysis, peritoneal macrophages and liver leukocytes were washed twice and suspended in PBS containing 0.1% BSA and 0.05% sodium azide. A total of 1 × 10^6^ cells per 100 µl were incubated with F4/80-PE-Cyanine 5 (eBioscience, CA, USA), CD206-FITC (eBioscience, CA, USA) and CD16/32-PE (eBioscience) for 30 min at 4 °C in the dark. After washing twice, the cells were pelleted and resuspended. Data were acquired on a FACScan flow cytometer (Beckman Coulter) and analysed using FlowJo software (Treestar, San Carlos, CA, USA).

### Statistical analysis

All statistical analyses were performed using SPSS 13.0 software (SPSS Inc., Chicago, IL, USA). Significant differences between groups were determined using one-way analysis of variance (ANOVA). When data could not satisfy the conditions of ANOVA, Kruskal-Wallis test was used. All data shown are presented as the mean ± SEM, and *P* values ≤ 0.05 were considered statistically significant.

## Results

### rSj16 protein and Sj16 peptide alleviate hepatic granuloma in *S. japonicum*-infected mice

To determine the protective effects of rSj16, *S. japonicum-*infected mice were treated with the rSj16 protein and Sj16 peptide at different time points post-infection; injection of the recombinant *S. japonicum* 28 KDa glutathione S-transferase (GST) protein was used in control animals. Treatment with either rSj16, Sj16 peptide or GST did not significantly affect the worm burden or egg counts in infected mice (data not shown). However, treatment of infected mice with rSj16 or Sj16 peptide at different time points post-infection resulted in a significant reduction in granuloma size compared with the infected untreated group (Fig. 1b, c). Infected animals treated with rSj16 demonstrated a marked decrease in granuloma formation (54%, 76% and 46%) at weeks 6, 8 and 10, respectively (week 6: *χ*^2^ = 30.275, *df* = 3, *P* < 0.0001; week 8: *χ*^2^ = 25.851, *df* = 3, *P* < 0.0001; week 10: *χ*^2^ = 16.231, *df* = 3, *P* = 0.001). On the other hand, animals treated with Sj16 peptide displayed 57%, 48% and 12% decreases in granuloma formation at weeks 6, 8 and 10, respectively (week 6: *χ*^2^ = 30.275, *df* = 3, *P* = 0.001; week 8: *χ*^2^ = 25.851, *df* = 3, *P* = 0.043; week 10: *χ*^2^ = 16.231, *df* = 3, *P* = 0.276). By contrast, administration of GST resulted in insignificant reductions (*P* > 0.05) in hepatic granuloma size at every time point post-infection compared with the infected untreated groups (Fig. 1b, c).

### Hepatic fibrosis is relieved after treatment with the rSj16 protein and Sj16 peptide

**Fig. 2:**
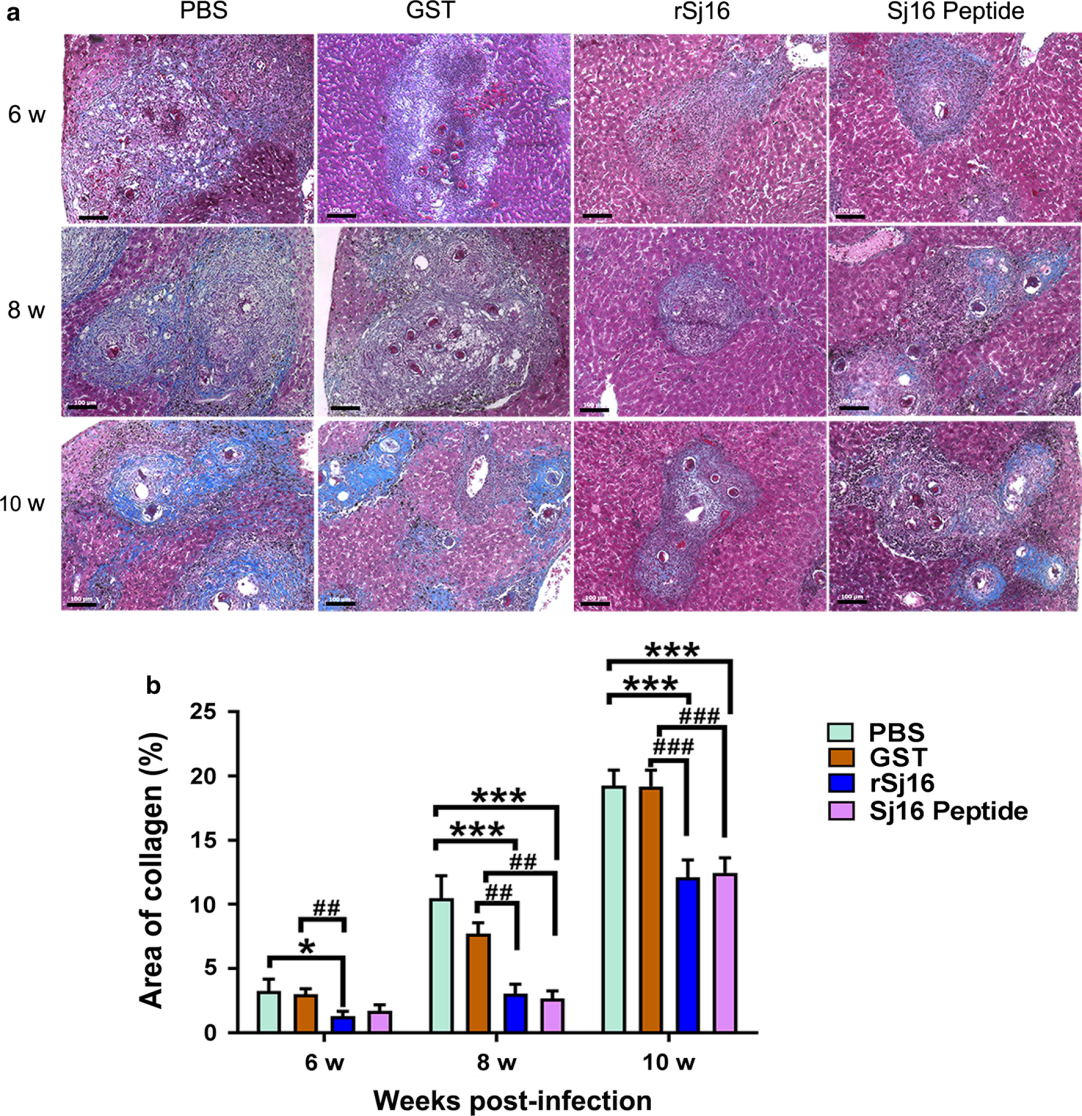
Hepatic fibrosis was relieved after treatment with rSj16 protein and Sj16 peptide. **a** Representative liver granulomas stained with Masson’s Trichrome at 6, 8 and 10 weeks post-infection (collagen in blue). *Scale-bars*: 100 μm. **b** Collagen deposition positive areas were digitized and analyzed on Image Pro Plus image software. Results are expressed as the mean ± SEM. *P < 0.05, **P < 0.01, ***P < 0.001, compared with the PBS group; ^##^*P* < 0.01, ^###^*P* < 0.001, compared with the GST group. Similar results were obtained in two repeat experiments

Hepatic fibrosis is the development of granulomas in the chronic phase, which subsequently cause serious complications. To prove whether Sj16 regulates tissue fibrogenesis, liver collagen deposition was measured with Masson’s trichrome staining. Strikingly, in agreement with the decreased granulomatous inflammation, treatment with rSj16 and Sj16 peptide showed a significant reduction in fibrosis compared with infected untreated mice, and treatment with rSj16 decreased the pathology by 60%, 71% and 36% at weeks 6, 8 and 10, respectively (Fig. 2; week 6: *χ*^2^ = 15.314, *df* = 3, *P* = 0.046; week 8: *F*_(3, 16)_ = 12.107, *P* < 0.0001; week 10: *F*_(3, 16)_ = 8.582, *P* = 0.001). Treatment with Sj16 peptide decreased the pathology by 47%, 74% and 34% at weeks 6, 8 and 10, respectively (Fig. 2; week 6: *χ*^2^ = 15.314, *df* = 3, *P* = 0.156; week 8: *F*_(3, 16)_ = 12.107, *P* < 0.0001; week 10: *F*_(3, 16)_ = 8.582, *P* = 0.001). However, by contrast, insignificant amelioration of liver fibrosis was observed in the GST-treated groups compared with the untreated groups (Fig. 2).

### The release of pro-inflammatory cytokines is prevented in infected mice after treatment with the rSj16 protein and the Sj16 peptide

**Fig. 3:**
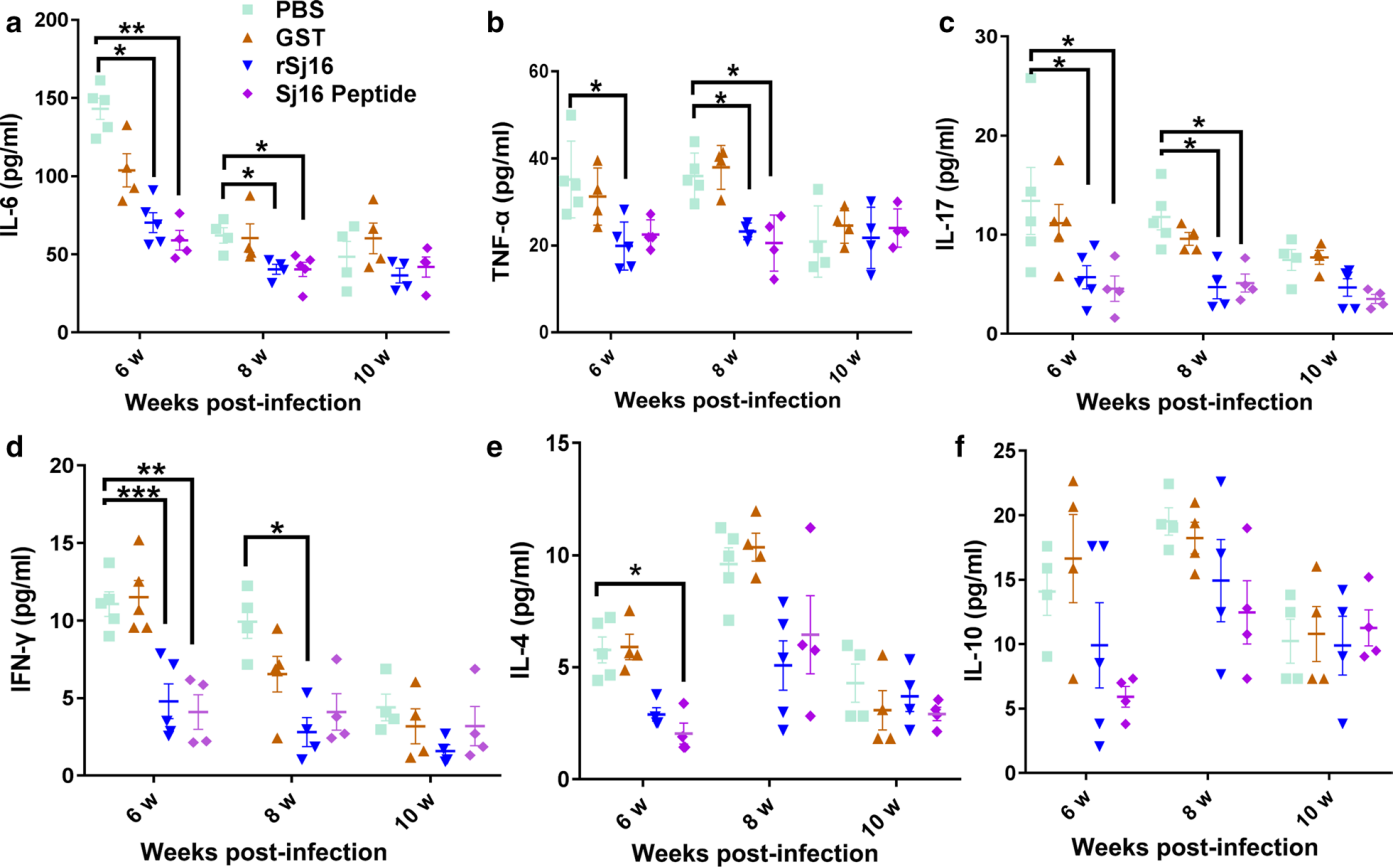
Pro-inflammatory cytokines in sera were inhibited after treatment with rSj16 protein and Sj16 peptide. Infected BALB/c mice were respectively injected with PBS, GST protein (50 µg/d), rSj16 (50 µg/d), Sj16 peptide (50 µg/d) for 7 consecutive days before the animals were sacrificed. Serum was obtained for cytokine expression analysis at 6, 8 and 10 weeks post-infection. Results are expressed as the mean ± SEM. Significant differences have been noted, *P < 0.05, **P < 0.01 and ***P < 0.001. Similar results were obtained in three repeat experiments

The cytokine-producing profile in serum was measured to investigate the effect of the Sj16 treatments on the systemic immune response, including pro-inflammatory cytokines (IL-6 and TNF-α), a Th1 cytokine (IFN-γ), a Th2 cytokine (IL-4), a Th17 cytokine (IL-17) and a regulatory cytokine (IL-10). Treatment with the rSj16 protein and Sj16 peptide drastically inhibited the increase in the levels of IL-6 (Fig. 3a,), TNF-α (Fig. 3b), IFN-γ (Fig. 3d), IL-17 (Fig. 3c) and IL-4 (Fig. 3e) production at weeks 6 and 8 in infected mice (Fig. 3, Additional file 1: Table S1). These results revealed that the anti-inflammatory effect of rSj16 was more effective in the early stages of administration because infected mice treated with rSj16 from week 9 to week 10 did not show reductions of serum cytokine production compared to infected untreated mice (*P* > 0.05). In addition, the regulatory cytokine IL-10 (Fig. 3f) also tended to be decreased by the rSj16 treatment, even though these effects were not statistically significant. However, the control group treated with GST did not show a significant change of any cytokine production at different time points in comparison with the infected control group (*P* > 0.05).

### Combined treatment with PZQ and Sj16 peptide effectively decreases granulomas and fibrosis formation as well as the production of inflammatory cytokines

**Fig. 4:**
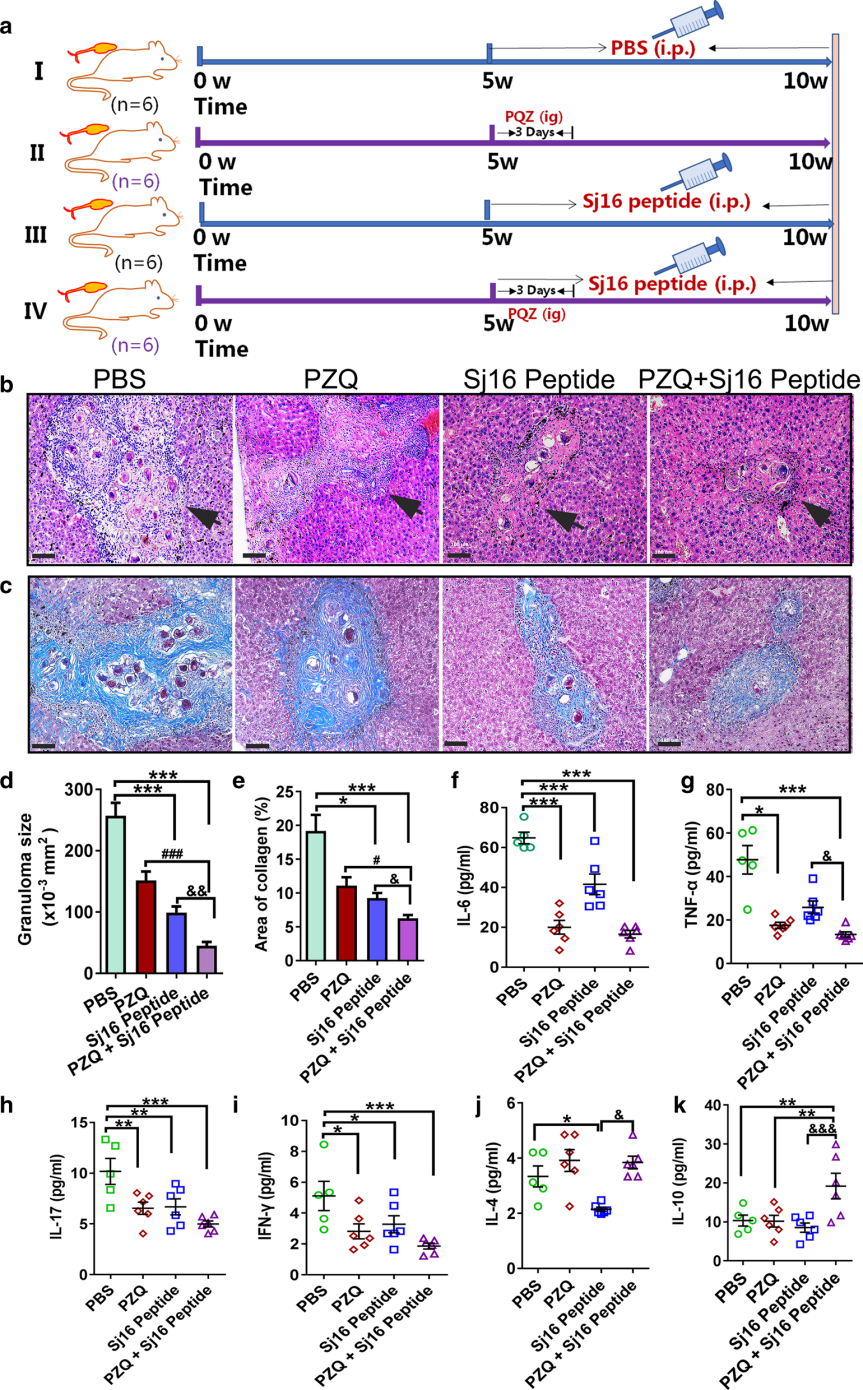
Compared the therapeutic effects of PZQ or PZQ combination with Sj16 peptide in infected mice. **a** The experiment flow chart. *Schistosoma japonicum*-infected mice were administered with praziquantel (PZQ) alone (150 mg/kg/day for 3 days), Sj16 peptide alone (50 µg/d for 5 weeks), or combined (PZQ: 150 mg/kg/day for 3 days; Sj16 peptide: 50 µg/d for 5 weeks), respectively, at week 5 post-infection, and sacrificed at 10 weeks post-infection. **b** H&E staining of representative hepatic granulomas. *Scale-bars*: 100 μm. **c** Representative liver granulomas stained with Masson’s Trichrome (collagen in blue). *Scale-bar*: 100 μm. **d** Analysis of granuloma size in the liver. **e** Analysis of fibrosis. **f–k** The production of cytokines in serum. Results are expressed as the mean ± SEM of 5–6 mice per group. *P < 0.05, **P < 0.01, ***P < 0.001, compared with the PBS group; ^##^*P* < 0.01, ^###^*P* < 0.001, the PZQ group compared with PZQ + Sj16 peptide group; ^&^*P* < 0 .05, ^&&^*P* < 0.01, ^&&&^*P* 0.001, the Sj16 peptide group compared with PZQ + Sj16 peptide group

To explore whether Sj16 combined with PZQ is superior to PZQ alone in the treatment of schistosomiasis, the therapeutic effects of these treatments on alleviating liver granulomatous inflammation and fibrosis were compared. We treated four groups of mice with PZQ, Sj16 peptide, PZQ + Sj16 peptide or vehicle PBS alone (infected untreated control group) after mice were infected with *S. japonicum* cercariae. The results showed that there were no significant differences in worm burden or egg counts between the PZQ alone group and the PZQ + Sj16 peptide group (Additional file 2: Table S2, *P* > 0.05). However, all of the treatments reduced the size of liver granulomas (Fig. 4b, d), fibrosis (Fig. 4c, e), and the pro-inflammatory cytokines IL-6 (Fig. 4f), TNF-α (Fig. 4g), IL-17 (Fig. 4h), the Th1 cytokine IFN-γ (Fig. 4i) compared to PBS treated mice (Fig. 4, Additional file 3: Table S3). Intriguingly, compared with administration of PZQ alone, the size of the granulomas drastically decreased by 70% in the PZQ + Sj16 peptide group (Fig. 4b, d; *χ*^2^ = 59.382, *df* = 3, *P* < 0.0001), and fibrosis decreased by 43% (Fig. 4c, e; *χ*^2^ = 27.941, *df* = 3, *P* = 0.02). The inflammatory cytokines IL-6, TNF-α, IL-17 and IFN-γ tended to be insignificantly reduced (*P* > 0.05). It is interesting to note that the production of IL-10 (Fig. 4k; *F*_(3, 19)_ = 5.585, *P* = 0.005) and IL-4 (Fig. 4j; *F*_(3, 19)_ = 8.1, *P* = 0.861) up-regulated after treatment with PZQ + Sj16 peptide, compared to the PZQ alone groups. Taken together, these data suggest that the combination treatment of PZQ and Sj16 could be of particular interest for treating schistosomiasis.

### rSj16 protein and Sj16 peptide alternatively activate M2 macrophages

**Fig. 5:**
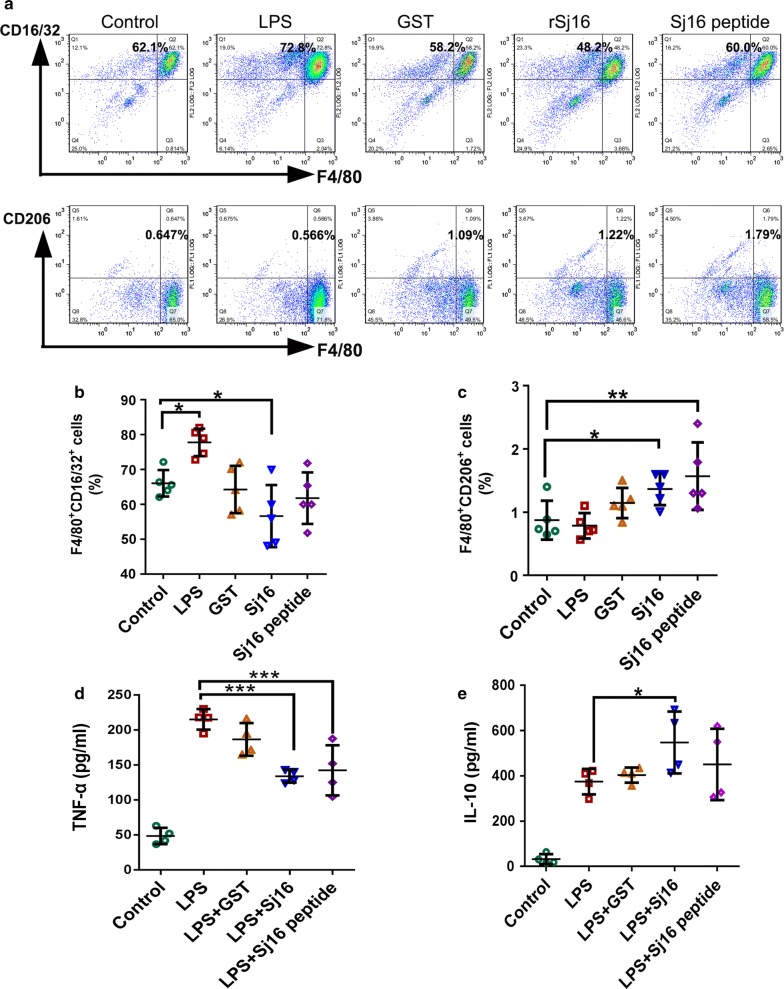
rSj16 protein and Sj16 peptide inhibited M1 macrophages and alternatively activated M2 macrophages *in vitro*. Peritoneal macrophages obtained from BABL/c mice were stimulated with LPS (1 μg/ml), GST (10 μg/ml), rSj16 (10 μg/ml), Sj16 peptide (10 µg/ml), or medium alone. Macrophages were harvested after 24 h. **a** The expression of CD16/32 (M1) and CD206 (M2) macrophages were evaluated by FCM analysis. **b** Percentages of F4/80^+^ CD16/32^+^ macrophages. **c** Percentages of F4/80^+^ CD206^+^ macrophages. **d** The expression of TNF-α. **e** The expression of IL-10. Results are expressed as the mean ± SEM of 3 independent experiments. Significant differences were detected, *P < 0.05

To illustrate the mechanisms of the action of Sj16, peritoneal macrophages isolated from normal BABL/c mice were stimulated with LPS, GST, rSj16, Sj16 peptide, or medium alone and then subjected to analysis for CD16/32 (a phenotypic marker for M1 macrophages) and CD206 (a phenotypic marker for M2 macrophages) [42] expression by flow cytometry. LPS was administered as the positive control to induce M1 macrophage activation. rSj16-treated macrophages showed a slight reduction in the percentage of M1 macrophages (Fig. 5b; *F*_(4, 20)_ = 7.274, *P* = 0.033) and a significant increase in the percentage of M2 macrophages (Fig. 5c; *F*_(4, 20)_ = 4.976, *P* = 0.028) compared with the untreated group (Fig. 5a, b). Interestingly, no significant difference in the percentage of the M1 subtype between Sj16 peptide-treated group and the control group was observed (Fig. 5a, b; *P* > 0.05). There was approximately a 2-fold increase in the percentage of M2 macrophages in the Sj16 peptide-treated group compared with that of the control group (Fig. 5c; *F*_(4, 20)_ = 4.976, *P* = 0.003). In addition, rSj16 and Sj16 peptide effectively downregulated LPS-induced TNF-α (a marker for M1 macrophages) expression (Fig. 5d; rSj16: *F*_(4, 15)_ = 35.525, *P* < 0.0001; Sj16 peptide: *F*_(4, 15)_ = 35.525, *P* < 0.0001) and upregulated IL-10 (a marker for M2 macrophages) expression (Fig. 5e; rSj16: *F*_(4, 15)_ = 15.853, *P* = 0.025; Sj16 peptide: *F*_(4, 15)_ = 15.853, *P* = 0.29), confirming that Sj16 can alternatively activate M2 macrophages *in vitro*.

**Fig. 6:**
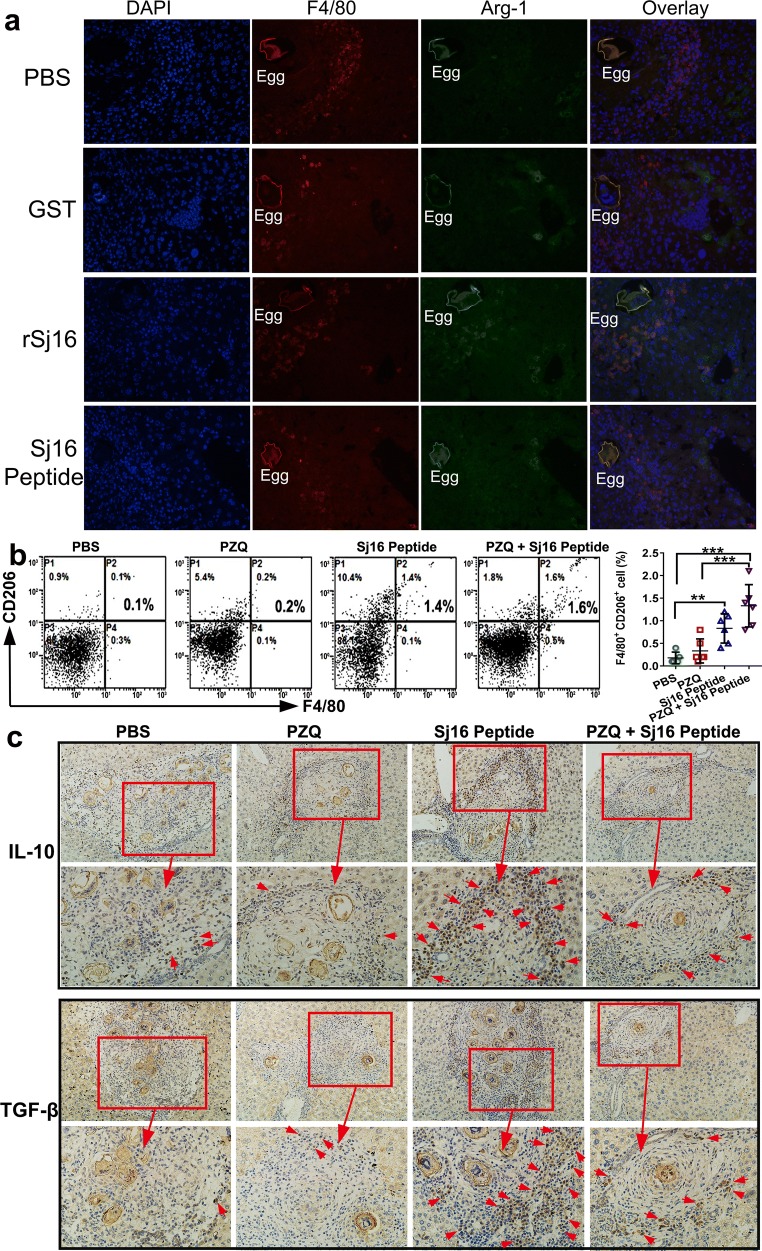
rSj16 protein and Sj16 peptide increased M2 macrophages in liver of *S. japonicum*-infected mice. **a** Sections taken from livers of *S. japonicum*-infected mice treated with PBS, GST protein (50 µg/d), rSj16 (50 µg/d), Sj16 peptide (50 µg/d) for 7 consecutive days before the animals sacrificed at 8 weeks post-infection. Co-immunostaining of F4/80 and Arg-1, photographed at 40×. **b** Liver leukocytes were harvested from 10 week *S. japonicum*-infected mice administered PZQ alone (150 mg/kg/day for 3 days), Sj16 peptide alone (50 µg/d for 5 weeks), or combined (PZQ: 150 mg/kg/day for 3 days; Sj16 peptide: 50 µg/d for 5 weeks) at week 5 week post-infection. The expression of F4/80^+^ CD206^+^ (M2) macrophages in the liver were evaluated by FCM analysis. **c** The expression of IL-10 and TGF-β in the liver was identified by immunohistochemistry using IL-10 and TGF-β antibody, respectively. Arrows indicate the positive signal. Results are expressed as the mean ± SEM of 2 independent experiments (*n* = 6). Significant differences were detected, *P < 0.05

Next, we compared the expression of arginase 1 (Arg-1), a M2-specific enzyme in macrophages, in the liver of GST, rSj16, Sj16 peptide, or vehicle (PBS)-treated infected mice using immunofluorescence. Macrophages were detected by staining with antibodies against F4/80. F4/80 positive cells (red fluorescence) were reduced around granuloma tissue of rSj16 and Sj16 peptide-treated mice in comparison with infected untreated mice (Fig. 6a). Meanwhile, a strong Arg-1 interaction (green fluorescence) and overlay (orange staining) were observed in the livers of mice treated with rSj16 and Sj16 peptide compared to infected untreated and GST-treated mice. These findings suggest that M2 macrophages are alternatively activated *in vivo* by the Sj16 treatment. Moreover, to provide further evidence, we analysed the expression of CD206 in liver leukocytes from infected mice treated with PZQ, Sj16 peptide, PZQ + Sj16 peptide and PBS. Remarkably, there were significant increases in the percentage of F4/80^+^CD206^+^ (4-fold) in the liver of Sj16 peptide (Fig. 6b; *F*_(3, 19)_ = 12.142, *P* = 0.003) and PZQ + Sj16 peptide-treated mice (> 8-fold, Fig. 5b; *F*_(3, 19)_ = 12.142, *P* < 0.0001) compared with the PBS group (Fig. 6b). Finally, the expression of the M2 macrophage markers IL-10 and TGF-β in the liver was analysed by immunohistochemistry. The results showed that the expression of IL-10 and TGF-β around egg granulomas in livers was obviously upregulated in Sj16 peptide and PZQ + Sj16 peptide-treated mice. Taken together, these results indicate that M2 macrophages are alternatively activated to attenuate hepatic granuloma formation and fibrosis by Sj16.

## Discussion

Potent pro-inflammatory responses along with granuloma formation during acute schistosomiasis and fibrosis as well as serious complications during the chronic phase are the primary causes of death in schistosomiasis [4]. Therefore, anti-inflammatory responses to limit excessive organ injury and anti-fibrosis development are necessary to prevent host death. To our knowledge, the results of the present study provide the first evidence that the anti-inflammatory protein rSj16 has significant protective effects in *S. japonicum*-infected mice, as shown by decreased granuloma formation, areas of collagen deposition, and inhibition of the pro-inflammatory response that underlies granuloma development. These results are in agreement with our previous data showing a potent anti-inflammatory effect of rSj16 in animal models of distinct diseases [32–35]. Remarkably, these protective effects are more visible when animals are treated with Sj16 during the early stages of infection (acute stage, week 6 and 8 post-infection), yet Sj16 has a weak inhibitory effect on granuloma and fibrosis at week 10 post-infection, without an obvious effect on the downregulation of cytokines. We suspect that this result occurs because the host regulates its own immune balance by upregulating Treg cells and by other means to reduce granulomatous inflammation at week 10 of *S. japonicum* infection; therefore, the effect of Sj16 cannot be clearly observed. Hence, the result suggests that the optimal time for administration of Sj16 is in the early stage of infection. PZQ kills the adult worms but have no significant effect on eggs, and these eggs will continue to induce fibrosis [29, 43]. Our results showed that rSj16 have no effects on worm burden or egg counts. However, protective anti-inflammatory and anti-fibrotic effects were observed following treatment of infected mice with PZQ + Sj16, which was much more efficacious than administration of PZQ alone. Surprisingly, this treatment may additionally trigger upregulation of an immunomodulatory cytokine, IL-10, which inhibits the production of pro-inflammatory cytokines [44, 45]. A previous study showed that GST from *S. mansoni* has protective effects when provided with an adjuvant to infected animals with schistosomiasis, including a reduction of the worm burden, egg counts, or extent of liver granuloma formation [46, 47]. However, this protective activity was not significant when *Sm*GST was administered alone before or a few weeks after infection with *S. mansoni*, as reported by Sun et al. [48]. Indeed, similar results were observed in our study.

We investigated the mechanism of action of Sj16 that led to its protective activity in the pathological and inflammatory responses and found that its activity was independent of its direct effects on the parasite burden and liver egg counts and was only attributed to its effects on immunological regulation. It is well known that immune cytokines are closely associated with the formation of egg-induced granulomas and fibrosis in schistosomiasis [12, 13, 49]. Interestingly, we found that treatment with Sj16 resulted in a drastic suppression of Th1, Th2 and Th17 cytokine production in *S. japonicum*-infected mice, including IFN-γ, TNF-α, IL-4, and IL-6. Prolonged Th2 and Th17 responses promote the development of hepatic granulomatous inflammation and fibrosis [11–13, 50]. Therefore, the marked inhibition of the Th2 and Th17 cytokine release in Sj16-treated mice observed in this study could be explained in part by the significant decreases in hepatic granuloma formation and fibrosis.

On the other hand, macrophages are among the main cellular constituents of granulomas [14] and play critical roles in regulating inflammation and fibrosis [23, 51, 52]. Our previous study revealed that Sj16 can decrease the levels of pro-inflammatory cytokines, such as IL-6 and TNF-α, and increase the levels of IL-10 in the murine macrophage cell line RAW264.7 [34]. Therefore, we hypothesized that the mechanisms of Sj16 attenuation of hepatic granulomatous inflammation and fibrosis in *S. japonicum*-infected mice might be related to the induction of macrophages toward M2 polarization. Notably, in this study, flow cytometry analysis revealed that CD206 expression, a marker associated with M2 polarization, was significantly increased after the Sj16 treatment in peritoneal macrophages and in livers leukocytes of mice infected with *S. japonicum*. Additionally, immunofluorescence analysis showed that Arg-1-positive macrophages were increased around granulomas in Sj16-treated mice. Interestingly, Herbert et al. [21] reported that M2 macrophages are essential for protecting against organ injury through downregulation of the Th1 response and egg-induced inflammation during *S. mansoni* infection. In addition, Pesce et al. [24] found that Arg-1-expressing macrophages function as inhibitors of granulomatous inflammation, fibrosis, and mortality in *S. mansoni*-infected mice. Furthermore, Kevin et al. [25] reported that distinct subsets of M2 macrophages controlled inflammation and fibrosis in chronic schistosomiasis, that the Arg-1-expressing M2 population slowed the development of lethal fibrosis and that Lyz2^hi^IL-4Rα^+^ M2 macrophages mediated the down-regulation of granulomatous inflammation in schistosomiasis. Accordingly, it is suggested that the mechanisms of Sj16 attenuation of hepatic granulomatous inflammation and fibrosis in *S. japonicum*-infected mice may be strongly linked to the induction of macrophages toward M2 polarization.

Interestingly, the egg-induced pathology of schistosomes was inhibited by their own excretory/secretory Sj16 due to the ability of this parasite to avoid attack by the host immune defence response. When the cercariae of the schistosome penetrate the skin of the host and migrate to the lungs and finally the liver and mesenteric veins to become mature for oviposition, they must face the hostʼs immune-defence mechanisms. Therefore, schistosomes have evolved highly effective strategies to downregulate the hostʼs immune response to facilitate their own development and transmission [11, 53, 54]. For example, skin-stage larvae induce localized production of pro-inflammatory cytokines (e.g. IL-1β, IL-12, TNF-α, IL-6) in the host as well as mediators with an immunoregulatory function (e.g. IL-10, prostaglandins E2 and D2) to inhibit host immune cell migration and activation [53, 55], which downregulate host immune responses and enhance the ability of schistosome larvae to exit the skin en route and reach the final sites for their maturation. In addition, some components of schistosome eggs have been shown to affect the maturation and activation of host immune cells by regulating the production of cytokines to dampen inflammatory responses to schistosome eggs, thereby preventing organ damage [11]. Thus, it appears that these immunoregulatory mechanisms of schistosomes not only promote parasite survival but also limit the excessive pathology that may result in the death of the host. Indeed, we showed that Sj16 is present in cercariae, adults and eggs of *S. japonicum*, with particularly high expression in cercariae and eggs (Additional file 4: Figure S1), suggesting that Sj16 plays an important role in the immune evasion of *S. japonicum*. Therefore, this study may be helpful for better understanding the mechanisms of schistosome-mediated immune modulation and the interaction between the host and parasite.

## Conclusions

In conclusion, these data demonstrate for the first time the anti-inflammatory and anti-fibrotic effects of Sj16 in schistosomiasis using a mouse model and point to the use of the combination of Sj16 with PZQ, which may be a viable and promising therapeutic method in the clinical setting to treat patients with *S. japonicum* infection. Moreover, this study provides a better understanding of the mechanisms of schistosome-mediated immune modulation and the host-parasite interaction.

## Supplementary information

**Additional file 1: Table S1.** Reporting significant results from statistical tests of the levels of cytokines in Fig. 3.

**Additional file 2: Table S2.** Effects on worm and egg burden in *S. japonicum*-infected mice treated with PZQ alone or combined with Sj16 peptide.

**Additional file 3: Table S3.** Reporting significant results from statistical tests in Fig. 4.

**Additional file 4: Figure S1.** The expression of Sj16 in *S. japonicum* at different life-cycle stages.

## Data Availability

The datasets supporting the conclusions of this article are available from the corresponding author on reasonable request.

## Abbreviations

Th1: T helper 1
PZQ: praziquantel
rSj16: recombinant protein Sj16
LPS: lipopolysaccharide
GST: glutathione S-transferase
SDS-PAGE: sodium dodecyl sulphate-polyacrylamide gel electrophoresis
i.p.: intraperitoneally
ig: intragastric injection
H&E: hematoxylin and eosin
FITC: fluorescein isothiocyanate
DAPI: 4,6-diamidino-2-phenylindole
CBA: cytometric bead array
