# Supplementary material for: Recombinant Sj16 protein with novel activity alleviates hepatic granulomatous inflammation and fibrosis induced by Schistosoma japonicum associated with M2 macrophages in a mouse model
Source: Parasit Vectors. 2019 Sep 23;12:457. doi: 10.1186/s13071-019-3697-z (PMC6755699; doi:10.1186/s13071-019-3697-z)
Supplement: Supplementary file 1 — Additional file 1: Table S1. Reporting significant results from statistical tests of the levels of cytokines in Fig. 3. [file 13071_2019_3697_MOESM1_ESM.docx]

**Additional file 1: Table S1.** Reporting significant results from statistical tests of the levels of cytokines in Fig. 3

| **Cytokines** | **rSj16 *vs* PBS** | | **Sj16 peptide *vs* PBS** | |
| --- | --- | --- | --- | --- |
|  | in week 6 | in week 8 | in week 6 | in week 8 |
| IL-6 | *χ*^2^ = 13.789, *df*  = 3, *P* = 0.032 | *F*_(3, 13)_ = 4.338, *P* = 0.023 | *χ*^2^ = 13.789, *df*  = 3, *P* = 0.006 | *F*_(3, 13)_ = 4.338, *P* = 0.018 |
| TNF-α | *χ*^2^ = 10.79, *df*  = 3, *P* = 0.031 | *χ*^2^ = 9.857, *df*  = 3, *P* = 0.015 | *P* > 0.05 | *χ*^2^ = 9.857, *df*  = 3, *P* = 0.031 |
| IFN-γ | *F*_(3, 15)_ = 11.893, *P* = 0.001 | *χ*^2^ = 9.599, *df*  = 3, *P* = 0.017 | *F*_(3, 15)_ = 11.893, *P* = 0.002 | *P* > 0.05 |
| IL-17 | *F*_(3, 15)_ = 3.667, *P* = 0.023 | *χ*^2^ = 12.776, *df*  = 3, *P* = 0.039 | *F*_(3, 15)_ = 3.667, *P* = 0.015 | *χ*^2^ = 12.776, *df*  = 3, *P* = 0.015 |
| IL-4 | *P* > 0.05 | *P* > 0.05 | *χ*^2^ = 12.592, *df*  = 3, *P* = 0.034 | *P* > 0.05 |
