# Supplementary material for: Recombinant Sj16 protein with novel activity alleviates hepatic granulomatous inflammation and fibrosis induced by Schistosoma japonicum associated with M2 macrophages in a mouse model
Source: Parasit Vectors. 2019 Sep 23;12:457. doi: 10.1186/s13071-019-3697-z (PMC6755699; doi:10.1186/s13071-019-3697-z)
Supplement: Supplementary file 2 — Additional file 2: Table S2. Effects on worm and egg burden in S. japonicum-infected mice treated with PZQ alone or combined with Sj16 peptide. [file 13071_2019_3697_MOESM2_ESM.docx]

**Additional file 2: Table S2.** Effects on worm and egg burden in *S. japonicum*-infected mice treated with PZQ alone or combined with Sj16 peptide

| Group | Total worms | No. of eggs found in liver (/g) ×10^5^ | |
| --- | --- | --- | --- |
| PBS | 4.2 ± 1.0 | | 22.97 ± 3.74 |
| PZQ | 0 | | 10.20 ± 3.41^*^ |
| Sj16 Peptide | 3.8 ± 1.8 | | 19.70 ± 4.98 |
| PZQ + Sj16 Peptide | 0 | | 7.05 ±2 .83^**^ |

Data are expressed as the means± SEM. Significant differences in characteristics were noted between groups. ^*^ *P* <0 .05, ^**^*P* < 0.01, compared with the PBS group.
