# Supplementary material for: Recombinant Sj16 protein with novel activity alleviates hepatic granulomatous inflammation and fibrosis induced by Schistosoma japonicum associated with M2 macrophages in a mouse model
Source: Parasit Vectors. 2019 Sep 23;12:457. doi: 10.1186/s13071-019-3697-z (PMC6755699; doi:10.1186/s13071-019-3697-z)
Supplement: Supplementary file 3 — Additional file 3: Table S3. Reporting significant results from statistical tests in Fig. 4. [file 13071_2019_3697_MOESM3_ESM.docx]

**Additional file 3: Table S3.** Reporting significant results from statistical tests in Fig. 4

| **Parameters** | **PZQ *vs* PBS** | **Sj16 peptide *vs* PBS** | **PZQ + Sj16 peptide *vs* PBS** |
| --- | --- | --- | --- |
| **Granuloma** | *χ*^2^ = 59.382, *df*  = 3, *P* = 0.068 | *χ*^2^ = 59.382, *df*  = 3, *P* < 0.0001 | *χ*^2^ = 59.382, *df*  = 3, *P* < 0.0001 |
| **Fibrosis** | *χ*^2^ = 27.941, *df*  = 3, *P* = 1.0 | *χ*^2^ = 27.941, *df*  = 3, *P* = 0.024 | *χ*^2^ = 27.941, *df*  = 3, *P* < 0.0001 |
| **IL-6** | *F*_(3, 19)_ = 36.063, *P* < 0.0001 | *F*_(3, 19)_ = 36.063, *P* < 0.0001 | *F*_(3, 19)_ = 36.063, *P* < 0.0001 |
| **TNF-α** | *F*_(3, 19)_ = 19.439, *P* < 0.0001 | *F*_(3, 19)_ = 19.439, *P* < 0.0001 | *χ*^2^ = 17.82, *df*  = 3, *P* = 0.001 |
| **IL-17** | *F*_(3, 19)_ = 7.456, *P* = 0.004 | *F*_(3, 19)_ = 7.456, *P* = 0.006 | *F*_(3, 19)_ = 7.456, *P* < 0.0001 |
| **IFN-γ** | *F*_(3, 19)_ = 5.302, *P* = 0.012 | *F*_(3, 19)_ = 5.302, *P* = 0.04 | *F*_(3, 19)_ = 5.302, *P* = 0.001 |
