# Supplementary material for: Recombinant Sj16 protein with novel activity alleviates hepatic granulomatous inflammation and fibrosis induced by Schistosoma japonicum associated with M2 macrophages in a mouse model
Source: Parasit Vectors. 2019 Sep 23;12:457. doi: 10.1186/s13071-019-3697-z (PMC6755699; doi:10.1186/s13071-019-3697-z)

**Additional file 4: Figure S1.** The expression of Sj16 in *S. japonicum* at different stages. Sj16 expression was detected by Western blotting with Sj16 polyclonal antibody. Protein suspension (1mg/ml) was prepared by cercariae released from Oncomelania hupensis, eggs and adults of *S. japonicum* collected from the liver of 45 days-infected mice.


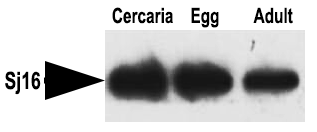

Supplement: Supplementary file 4 — Additional file 4: Figure S1. The expression of Sj16 in S. japonicum at different life-cycle stages. [file 13071_2019_3697_MOESM4_ESM.docx]
